# Supplementary material for: Loss of HIV candidate vaccine efficacy in male macaques by mucosal nanoparticle immunization rescued by V2-specific response
Source: Nat Commun. 2024 Oct 22;15:9102. doi: 10.1038/s41467-024-53359-2 (PMC11496677; doi:10.1038/s41467-024-53359-2)
Supplement: Supplementary file 1 — Supplementary Information [file 41467_2024_53359_MOESM1_ESM.pdf]

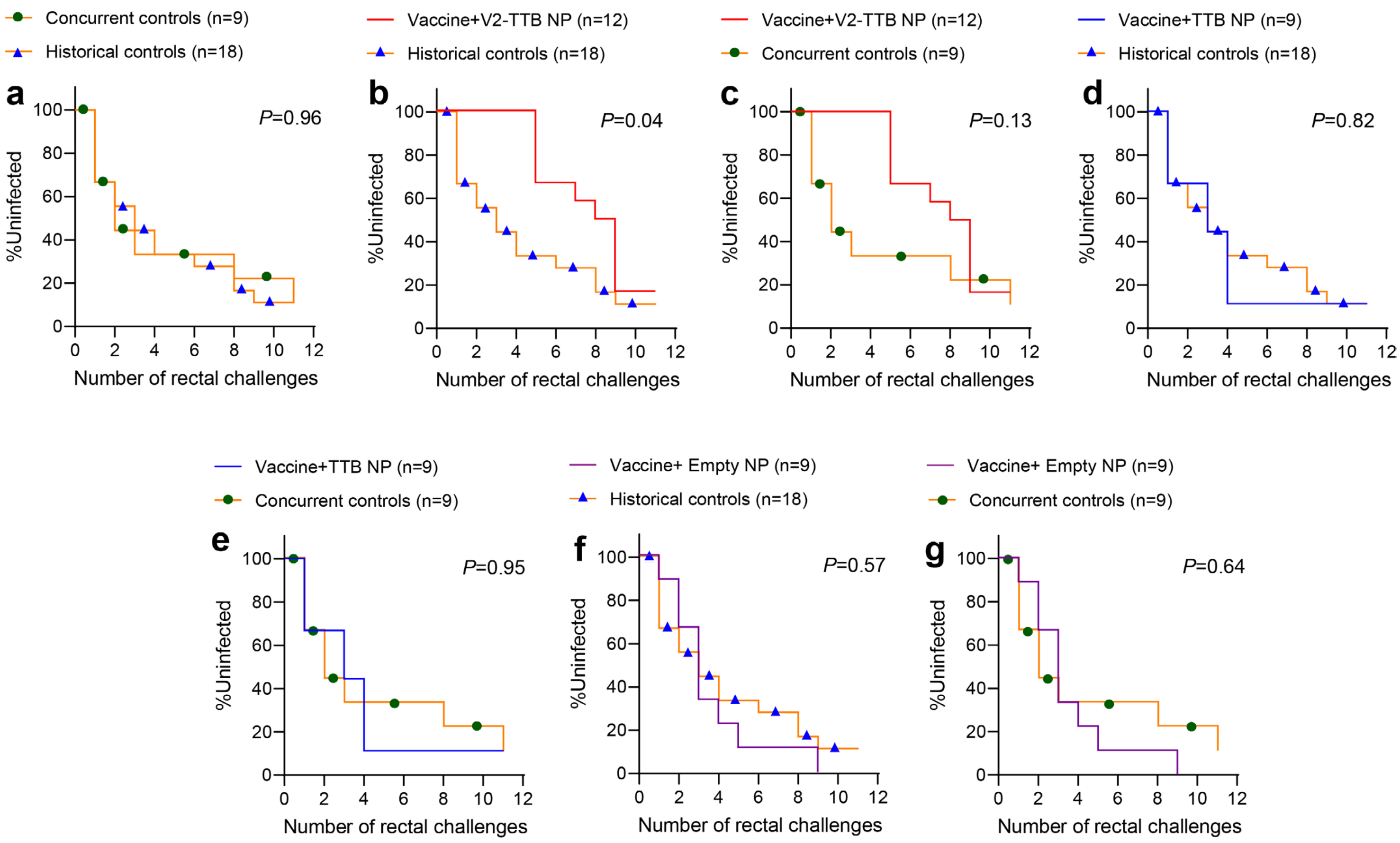

Supplementary Figure 1. Comparison of infection rate in different animal groups. a) No differences in delayed acquisition in the concurrent control group (n=9) were observed compared to the historical controls (n=18). **b**) Significant protection was observed in the V2-TTB NP group (n=12) compared to historical controls (n=18). **c**) A trend of protection was observed for V2-TTB NP group (n=12) compared to concurrent controls (n=9). **d, e**) Protection was not observed in the TTB NP group (n=9) compared to **d**) historical (n=18) or **e**) concurrent controls (n=9). **f, g**) No protection was observed in the empty NP group (n=9) compared to **f**) historical (n=18) or **g**) concurrent controls (n=9). Data shown in (**a-g**) were analyzed with log-rank (Mantel–Cox) test. Source data are provided as a Source Data file. Here, the orange, black, red, blue, and purple symbols represent the following groups: male naïve control, male macaques immunized with a systemic vaccine, vaccine combined with V2-TTB NP, vaccine combined with TTB NP, and vaccine combined with empty NP, respectively.

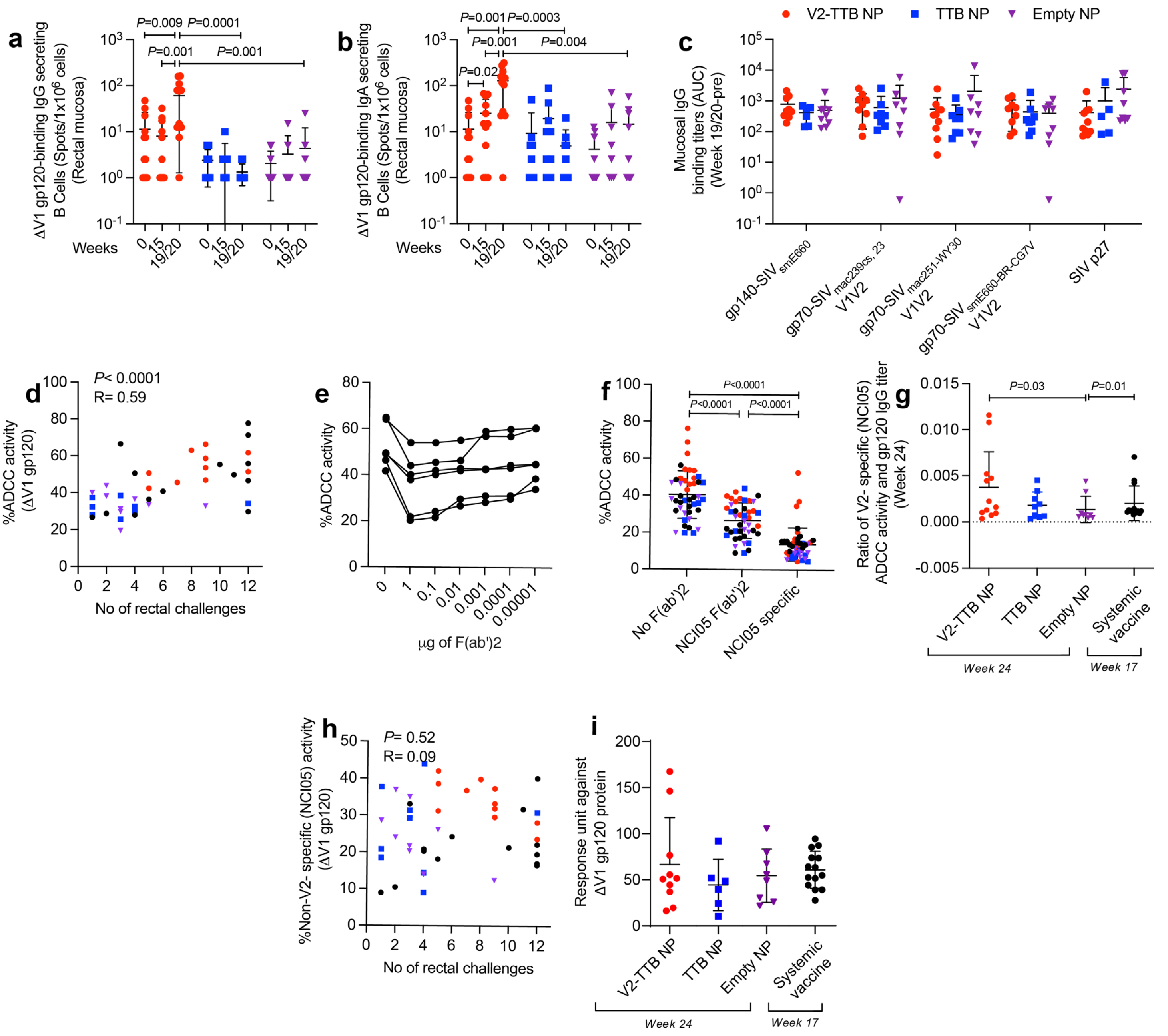

Supplementary Figure 2. Evaluation of humoral responses in plasma and mucosa in rhesus macaques. a, b) ΔV1 gp120-specific **a**) IgG (V2-TTB NP, n=12; TTB NP, n=9; empty NP, n=9) and **b**) IgA (V2-TTB NP, n=12; TTB NP, n=9; empty NP, n=9) producing antibody secreting B cells in rectal mucosa. **c**) Comparison of mucosal IgG binding titer (AUC) against different SIV immunogens in all groups of animals (V2-TTB NP, n=12; TTB NP, n=9; empty NP, n=9). **d**) Correlation of ADCC activity with number of intrarectal challenges in all animal groups combined (n=44). **e**) ADCC activity in the presence of different concentrations of F(ab')2 in systemic vaccinated animals (n=6). **f, g**) Comparison of **f**) total ADCC, non-V2 specific (NCI05) ADCC and V2-specific (NCI05) ADCC activity (n=44) and **g**) ratio of V2-specific ADCC to total gp120 specific IgG (n=44) in all group of animals irrespective to their vaccination regimen. **h**) Correlation of non-V2 specific (NCI05) ADCC in all groups of animals irrespective to their vaccination regimen (n=44). **i**) Comparison of antibody response units against ΔV1 gp120 in different groups of animals, where higher RU values indicated stronger binding of antibody to antigen (V2-TTB NP, n=12; TTB NP, n=9; empty NP, n=9; systemic vaccine, n=14). Data shown in (**a-c, f, g, i**) were analyzed with the two-sided Mann-Whitney test. Data shown in (**d, h**) were analyzed with the two-sided Spearman correlation test. Horizontal and vertical bars denote mean and SD. Source data are provided as a Source Data file. Here, the black, red, blue, and purple symbols represent male macaques immunized with a systemic vaccine, vaccine combined with V2-TTB NP, vaccine combined with TTB NP, and vaccine combined with empty NP, respectively.

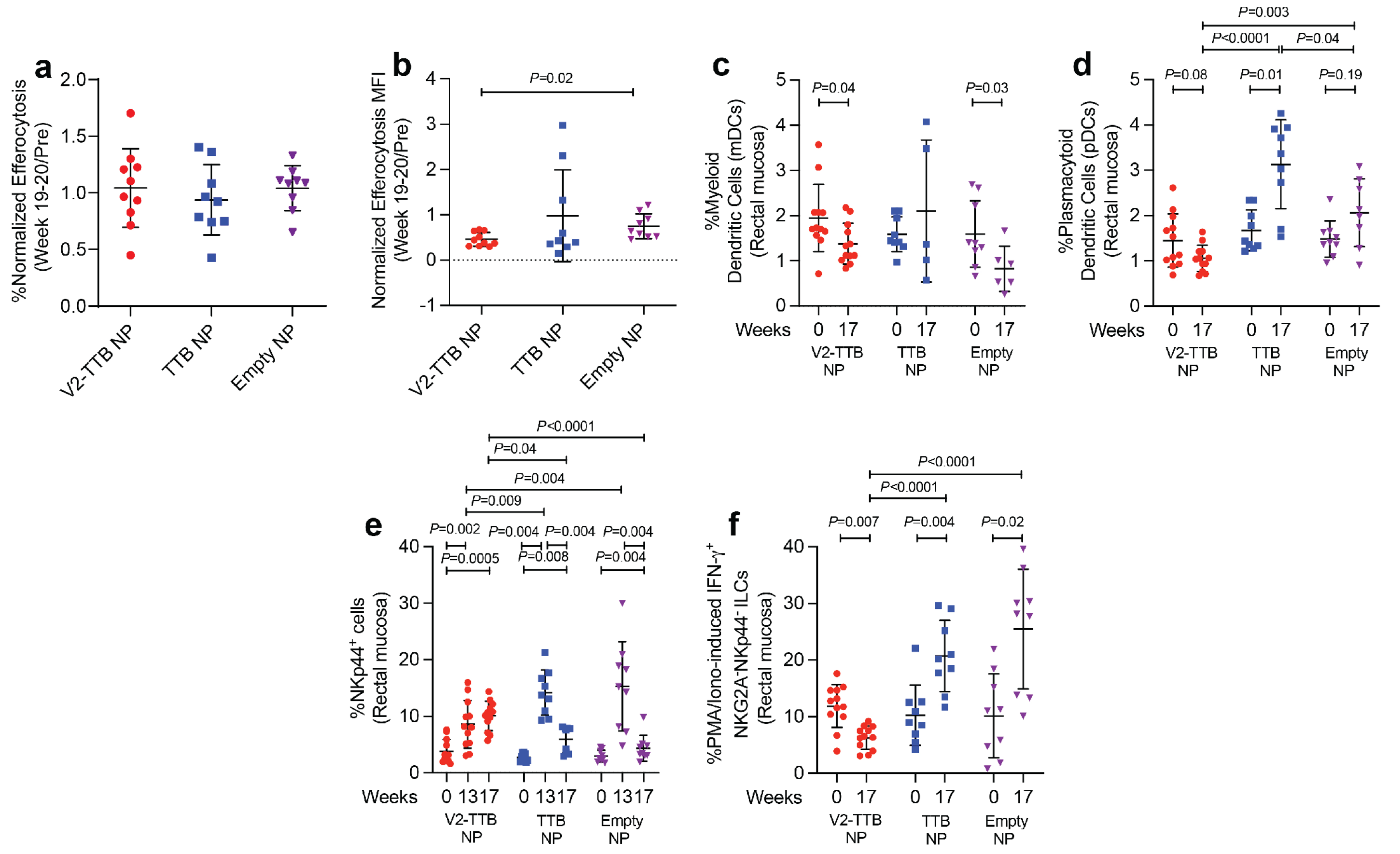

Supplementary Figure 3. Assessment of different subsets of systemic monocytes activity as well as mucosal dendritic cells, and mucosal NK/ILCs in the rectal mucosa of rhesus macaques. a-f) Comparison of **a**) percentage of efferocytosis (V2-TTB NP, n=10; TTB NP, n=9; empty NP, n=9), **b**) efferocytosis MFI (V2-TTB NP, n=10; TTB NP, n=9; empty NP, n=9) , **c**) mDCs (V2-TTB NP, n=12; TTB NP, n=5; empty NP, n=6), **d**) pDCs (V2-TTB NP, n=12; TTB NP, n=9; empty NP, n=9), **e**) $NKp44^{+}$ ILCs (V2-TTB NP, n=12; TTB NP, n=9; empty NP, n=9), and **f**) PMA/Ionomycin-induced IFN-γ^{+} $NKG2A^{-}NKp44^{-}$ ILCs (V2-TTB NP, n=12; TTB NP, n=9; empty NP, n=9) in different groups over the course of the study. Comparison of Data shown in (**a-f**) were analyzed with the two-sided Wilcoxon signed-rank test or two-sided Mann-Whitney test. Horizontal and vertical bars denote mean and SD. Source data are provided as a Source Data file. Here, the black, red, blue, and purple symbols represent male macaques immunized with a systemic vaccine, vaccine combined with V2-TTB NP, vaccine combined with TTB NP, and vaccine combined with empty NP, respectively.

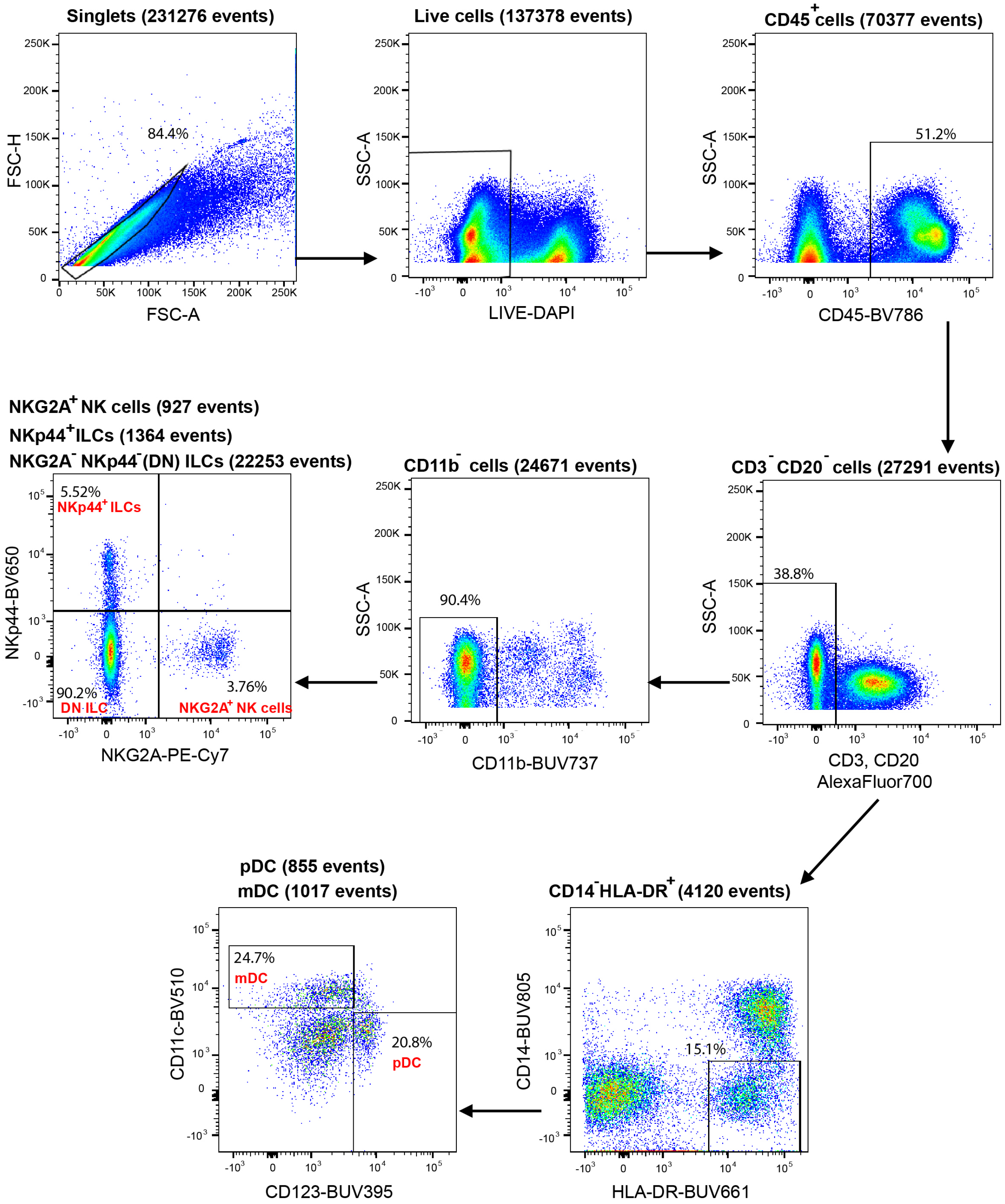

Supplementary Figure 4. Representative flow cytometry gating strategy. Gating strategy of mucosal dendritic cells and NK/innate lymphoid cells (ILCs). Each plot shows the frequency as well as the number of each cell population. Any cell of interest with less than 300 events was excluded from the analysis.

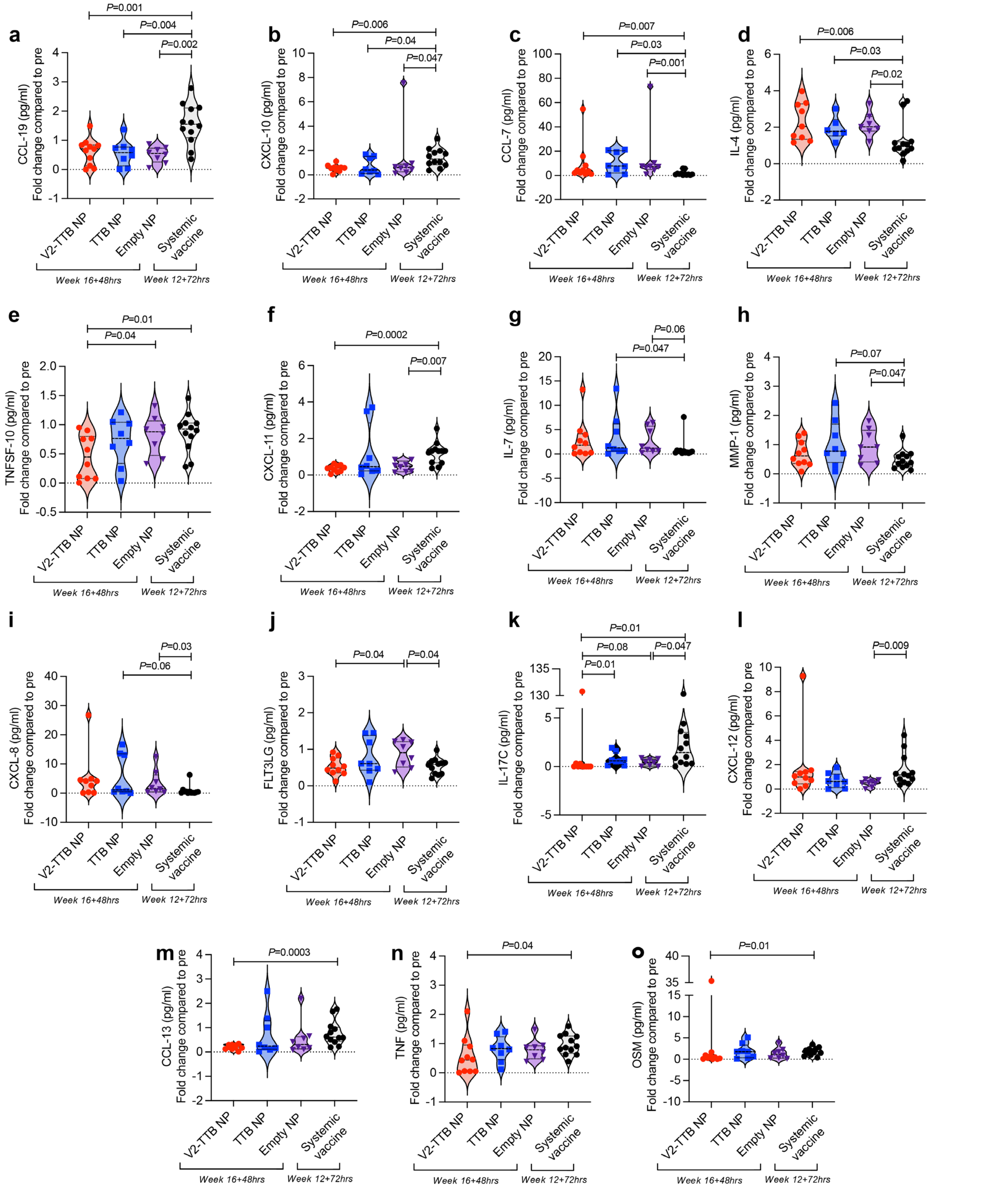

Supplementary Figure 5. Assessment of proteomic signature in the plasma of vaccinated animals. a-o) Comparison of plasma cytokine/chemokine fold-change relative to baseline of NP-treated vaccinated animals at 48hrs after last immunization (week 16 + 48 hrs) and systemically vaccinated animals at 72hrs after last immunization (week 12 + 72 hrs). Comparison of fold-change of **a**) CCL-19, **b**) CXCL-10, **c**) CCL-7, **d**) IL-4, **e**) TNFSF-10, **f**) CXCL-11, **g**) IL-7, **h**) MMP-1, **i**) CXCL-8, **j**) FLT3LG, **k**) IL-17C, **l**) CXCL-12, **m**) CCL-13, **n**) TNF and **o**) OSM in different animal groups (V2-TTB NP, n=10; TTB NP, n=8; empty NP, n=8; systemic vaccine, n=12). Violin plot data shown in (**a-o**) was analyzed with the two-sided Mann-Whitney test. Vertical bars denote median and quartiles. Source data are provided as a Source Data file. Here, the black, red, blue, and purple symbols represent male macaques immunized with a systemic vaccine, vaccine combined with V2-TTB NP, vaccine combined with TTB NP, and vaccine combined with empty NP, respectively.

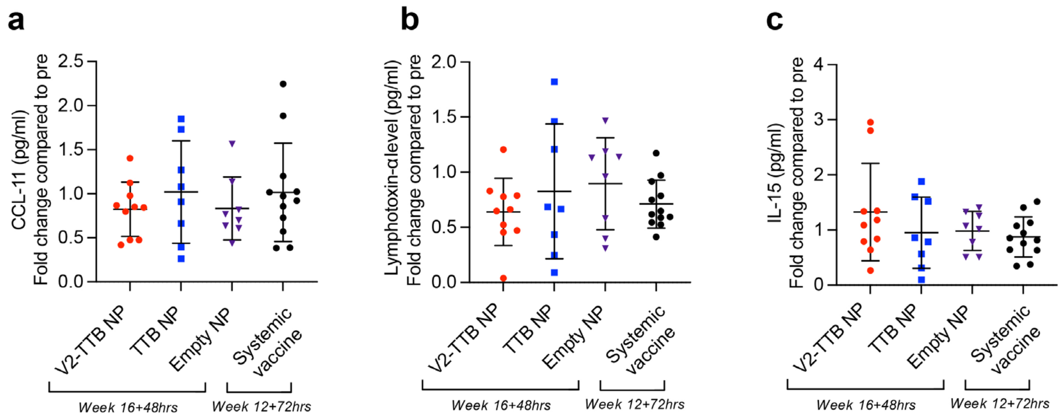

Supplementary Figure 6. Comparison of proteomic signature in the plasma of vaccinated animals. a-c) Comparison of plasma cytokine/chemokine fold-change relative to baseline of NP-treated vaccinated animals at 48 hrs after last immunization (week 16 + 48 hrs) and systemically vaccinated animals at 72 hrs after last immunization (week 12 + 72 hrs). Comparison of fold-change of **a**) CCL-10, **b**) LTα, **c**) IL-15 in different animal groups (V2-TTB NP, n=10; TTB NP, n=8; empty NP, n=8; systemic vaccine, n=12). Data shown in (**a-c**) were analyzed with the two-sided Mann-Whitney test. Horizontal and vertical bars denote mean and SD. Source data are provided as a Source Data file. Here, the black, red, blue, and purple symbols represent male macaques immunized with a systemic vaccine, vaccine combined with V2-TTB NP, vaccine combined with TTB NP, and vaccine combined with empty NP, respectively.
