# Supplementary material for: Loss of HIV candidate vaccine efficacy in male macaques by mucosal nanoparticle immunization rescued by V2-specific response
Source: Nat Commun. 2024 Oct 22;15:9102. doi: 10.1038/s41467-024-53359-2 (PMC11496677; doi:10.1038/s41467-024-53359-2)
Supplement: Supplementary file 3 — Reporting Summary [file 41467_2024_53359_MOESM3_ESM.pdf]

nature portfolio

Corresponding author(s): Genoveffa Franchini, Jay Berzofksy

Last updated by author(s): Sep 26, 2024

Reporting Summary

Nature Portfolio wishes to improve the reproducibility of the work that we publish. This form provides structure for consistency and transparency in reporting. For further information on Nature Portfolio policies, see our Editorial Policies and the Editorial Policy Checklist.

Statistics

For all statistical analyses, confirm that the following items are present in the figure legend, table legend, main text, or Methods section.

n/a	Confirmed	
☐	☒	The exact sample size (*n*) for each experimental group/condition, given as a discrete number and unit of measurement
☐	☒	A statement on whether measurements were taken from distinct samples or whether the same sample was measured repeatedly
☐	☒	The statistical test(s) used AND whether they are one- or two-sided<br>*Only common tests should be described solely by name; describe more complex techniques in the Methods section.*
☐	☒	A description of all covariates tested
☐	☒	A description of any assumptions or corrections, such as tests of normality and adjustment for multiple comparisons
☐	☒	A full description of the statistical parameters including central tendency (e.g. means) or other basic estimates (e.g. regression coefficient) AND variation (e.g. standard deviation) or associated estimates of uncertainty (e.g. confidence intervals)
☐	☒	For null hypothesis testing, the test statistic (e.g. *F*, *t*, *r*) with confidence intervals, effect sizes, degrees of freedom and *P* value noted<br>*Give P values as exact values whenever suitable.*
☒	☐	For Bayesian analysis, information on the choice of priors and Markov chain Monte Carlo settings
☐	☒	For hierarchical and complex designs, identification of the appropriate level for tests and full reporting of outcomes
☐	☒	Estimates of effect sizes (e.g. Cohen's *d*, Pearson's *r*), indicating how they were calculated

Our web collection on statistics for biologists contains articles on many of the points above.

Software and code

Policy information about availability of computer code

Data collection	FACSymphony A5 and examined using FACSDiva software (BD Biosciences).<br>Zeiss Axiocam System and Openlab software (Inprovision)<br>Molecular Devices E-max plate reader (San Jose, California, USA)<br>Droplet digital PCR
Data analysis	Prism 10 for macOS, Version 10.2.3 (347), April 21, 2024<br>FlowJo v10.10 (TreeStar, Inc.).<br>Microsoft Excel for Mac, 16.85 (24051214)

For manuscripts utilizing custom algorithms or software that are central to the research but not yet described in published literature, software must be made available to editors and reviewers. We strongly encourage code deposition in a community repository (e.g. GitHub). See the Nature Portfolio guidelines for submitting code & software for further information.

Data

Policy information about availability of data

All manuscripts must include a data availability statement. This statement should provide the following information, where applicable:

- Accession codes, unique identifiers, or web links for publicly available datasets
- A description of any restrictions on data availability
- For clinical datasets or third party data, please ensure that the statement adheres to our policy

All data used to generate the graphs in the manuscript and supplemental materials are available in the "Source Data" file.

Research involving human participants, their data, or biological material

Policy information about studies with human participants or human data. See also policy information about sex, gender (identity/presentation), and sexual orientation and race, ethnicity and racism.

Reporting on sex and gender	Research was not conducted in human participants
Reporting on race, ethnicity, or other socially relevant groupings	Research was not conducted in human participants
Population characteristics	Research was not conducted in human participants
Recruitment	Research was not conducted in human participants
Ethics oversight	Research was not conducted in human participants

Note that full information on the approval of the study protocol must also be provided in the manuscript.

Field-specific reporting

Please select the one below that is the best fit for your research. If you are not sure, read the appropriate sections before making your selection.

☒ Life sciences ☐ Behavioural & social sciences ☐ Ecological, evolutionary & environmental sciences

For a reference copy of the document with all sections, see nature.com/documents/nr-reporting-summary-flat.pdf

Life sciences study design

All studies must disclose on these points even when the disclosure is negative.

Sample size	The sample size of each group was determined by consultation with our statistician. The studies were not powered to compare SIVmac251 viral acquisition following viral exposure between vaccinated groups but rather between vaccinated and control groups.<br>If the animals are given up to 11 viral challenges and the numbers of challenges to infection are compared using the score test of the proportional hazards model (equivalent to log rank test), then at the two-sided P≤0.05 level with infection rate of 0.108 between the vaccine group of 12 or 9 animals and the control group of 27 animals, the power of the test is expected to be 35.3% (vaccine efficacy 44.8%) and 27.4% (vaccine efficacy 43.7%), respectively. With an infection rate of 0.065 between the vaccine group of 12 or 9 animals and the control group of 27 animals, the power of the test is expected to be 82.5% (vaccine efficacy 66.9%) and 71.5% (vaccine efficacy 66.4%), respectively. With an assumption of lower infection rate for the combination of vaccine and nanoparticles, a group size of 12 or 9 animals were added to different arms of the vaccine groups. The power calculations and the averaged vaccine efficacies (VE) are based on 10,000 simulated datasets where the infection rates in the historical control and the historical vaccine are 0.21 and 0.09, respectively.
Data exclusions	No data were excluded initially. However, based on the reviewers' request, any mucosal cell of interest with fewer than 300 events was excluded from the analysis.
Replication	The nature of the samples analyzed in the present studies, the limited amount of each sample collected from each animal and the cost of the non-human primate studies do not allow us to replicate the experiments. In the reported assays the replicates are represented by each animal enrolled in the study. All the data have been obtained with validated assays that have been used in previous publish work.
Randomization	39 macaques were randomized into 4 groups based on their weight and age : VV2-TTB NP+vaccine group (12 macaques); TTB NP+vaccine group (9 macaques); Empty NP+vaccine group (9 macaques); and control group (9 macaques).<br>For each in vitro study, samples from each animals received all of the different stimulations.
Blinding	The animal handlers were blinded to the vaccine groups. Always as possible, the investigators were blinded for in vitro assays. The data analyses were unblinded.<br>The authors compared the immune responses between different group of animals and for that unblinded was necessary.<br>All in vitro study was performed blinded. During data analysis, such as correlation and comparison of different group responses, the data were unblinded.

Reporting for specific materials, systems and methods

We require information from authors about some types of materials, experimental systems and methods used in many studies. Here, indicate whether each material, system or method listed is relevant to your study. If you are not sure if a list item applies to your research, read the appropriate section before selecting a response.

Materials & experimental systems

n/a	Involved in the study
☐	☒ Antibodies
☒	☐ Eukaryotic cell lines
☒	☐ Palaeontology and archaeology
☐	☒ Animals and other organisms
☒	☐ Clinical data
☒	☐ Dual use research of concern
☒	☐ Plants

Methods

n/a	Involved in the study
☒	☐ ChIP-seq
☐	☒ Flow cytometry
☒	☐ MRI-based neuroimaging

Antibodies

Antibodies used

Rectal mucosal NK/ILC, monocyte and dendritic cell phenotyping
Live/Dead blue dye (cat. #L34962, 0.5 µl) from Thermo Fisher,
Alexa 700 anti-CD3 (SP34-2; cat. #557917, 5µl), Alexa 700 anti-CD20 (2H7; cat. #560631, 5µl), BV510 anti-CD11c (3.9; cat. #748269, 5µl), BV650 anti-NKp44 (P44-8; cat. #744302, 5µl), BV786 anti-CD45 (D058-1283; cat. #563861,5 µl), BUV395 anti-CD123 (7G3; cat. #564195, 5µl), BUV496 anti-CD16 (3G8; cat. #612944, 5µl), BUV661 anti-HLA-DR (G46-6; cat. #612980, 5µl), BUV805 anti-CD14 (M5E2; cat. #565779, 5µl), from BD Biosciences (San Jose, California, USA); PE-Cy7 anti-NKG2A (Z199; cat. no. B10246, 5 µl) from Beckman Coulter and APC-Cy7 anti-CD11b (ICRF44; cat. #47-0118-42, 5µl) from Thermo Fisher (Waltham, MA, USA) .

Rectal mucosal NK/ILC cytokine expression upon gp120 peptides/PMA stimulation in vaccinated animals.
Live/Dead blue dye (cat. #L34962, 0.5 µl) from Thermo Fisher,
Alexa 700 anti-CD3 (SP34-2; cat. #557917, 5µl), Alexa 700 anti-CD20 (2H7; cat. #560631, 5µl), BV650 anti-NKp44 (P44-8; cat. #744302, 5µl), BV786 anti-CD45 (D058-1283; cat. #563861,5 µl), from BD Biosciences (San Jose, California, USA); PE-Cy7 anti-NKG2A (Z199; cat. no. B10246, 5 µl) from Beckman Coulter and APC-Cy7 anti-CD11b (ICRF44; cat. #47-0118-42, 5µl) from Thermo Fisher (Waltham, MA, USA).

BV421 anti-IFN-γ (B27; cat. #562988, 5µl) from BD Biosciences and PE-Cy5.5 anti-IL-17 (BL168; cat. # 512314, 5µl) from BioLegend (San Diego, California, USA).

CD4+ T-cell phenotypes
LIVE/DEAD™ Fixable Blue Dead Cell Stain (cat. #L23105, Thermo Fisher); Alexa 700 anti-CD3 (SP34-2; cat. #557917, 5µl), BV785 anti-CD4 (L200; cat. #563914, 5µl), PeCy5 anti-CD95 (DX2; cat. #559773, 5µl), BV650 anti-CCR5 (3A9; cat. #564999, 5µl), BUV496 anti-CD8 (RPA-T8; cat. #564804, 5µl), and FITC anti-Ki67 (B56; cat. #556026, 5µl) from BD Biosciences; APC Cy7 anti-CXCR3 (G025H7; cat. #353722, 5µl), and BV605 anti-CCR6 (G034E3; cat. #353420, 5µl), from BioLegend; and APC anti-α4β7, provided by the NIH Nonhuman Primate Reagent Resource

Validation

Marker Clone Validated website
1 CD3 (SP34-2) https://www.citeab.com/antibodies/2412955-557757-bd-pharmingen-apc-cy-7-mouse-anti-human-cd3?des=080d3bb9991f1653
2 CD20 (2H7) https://www.bdbiosciences.com/en-us/products/reagents/flow-cytometry-reagents/research-reagents/single-color-antibodies-ruo/pe-cf594-mouse-anti-human-cd20.562295
3 CD11c (3.9) doi: 10.3389/fimmu.2019.00779
4 NKp44 (P44-8) https://www.biolegend.com/en-us/products/apc-anti-human-cd336-nkp44-antibody-3850
5 CD45 (D058-1283) https://www.bdbiosciences.com/en-us/products/reagents/flow-cytometry-reagents/research-reagents/single-color-antibodies-ruo/pe-mouse-anti-nhp-cd45.552833
6 CD123 (7G3) doi: 10.3389/fimmu.2019.00779
7 CD16 (3G8) https://www.thermofisher.com/us/en/home/life-science/cell-analysis/cell-analysis-learning-center/cell-analysis-resource-library/ebioscience-resources/human-antibody-cross-reactivity-chart.html
8 HLA-DR (G46-6) https://www.fishersci.com/shop/products/hla-dr-mouse-anti-human-rhesus-cynomolgus-baboon-r718-clone-g46-6-also-known-as-l243-bd-horizon/p-7227060
9 CD14 (M5E2) https://www.bdbiosciences.com/en-us/products/reagents/flow-cytometry-reagents/research-reagents/single-color-antibodies-ruo/apc-mouse-anti-human-cd14.561383
10 NKG2A (Z199) https://www.beckman.com/reagents/coulter-flow-cytometry/antibodies-and-kits/single-color-antibodies/cd159a/a60797
11 CD11b (ICRF44) https://www.thermofisher.com/us/en/home/life-science/cell-analysis/cell-analysis-learning-center/cell-analysis-resource-library/ebioscience-resources/human-antibody-cross-reactivity-chart.html
12 IL-17 (BL168) https://www.nature.com/articles/s41564-020-00841-4
13 IFN-γ (B27) https://www.bdbiosciences.com/en-us/products/reagents/immunoassay-reagents/purified-mouse-anti-human-

ifn.554699
14 CD4 (L200) https://www.bdbiosciences.com/en-us/products/reagents/flow-cytometry-reagents/research-reagents/single-color-antibodies-ruo/fitc-mouse-anti-human-cd4.550628
15 CD95 (DX2) https://www.thermofisher.com/us/en/home/life-science/cell-analysis/cell-analysis-learning-center/cell-analysis-resource-library/ebioscience-resources/human-antibody-cross-reactivity-chart.html
16 CXCR5 (3A9) https://www.bdbiosciences.com/en-us/products/reagents/flow-cytometry-reagents/research-reagents/single-color-antibodies-ruo/bv650-mouse-anti-human-cd195.564999
17 CD8 (RPA-T8) https://www.thermofisher.com/us/en/home/life-science/cell-analysis/cell-analysis-learning-center/cell-analysis-resource-library/ebioscience-resources/human-antibody-cross-reactivity-chart.html
18 Ki67 (B56) https://www.nhpreagents.org/ReactivityDatabase
19 CXCR3 (G025H7) https://www.biolegend.com/en-us/products/biotin-anti-human-cd183-cxcr3-antibody-13320
20 CCR6 (G034E3) https://www.biolegend.com/en-us/products/purified-anti-human-cd196-ccr6-antibody-7512
21 α4β7 (A4B7R1) https://www.nhpreagents.org/Store/CategoryID/1/ProductID/37

Animals and other research organisms

Policy information about studies involving animals; ARRIVE guidelines recommended for reporting animal research, and Sex and Gender in Research

Laboratory animals	Thirty-nine Indian rhesus macaques obtained from the free-range breeding colony on Morgan Island, South Carolina, were used in this study. The macaques, aged 3 to 4 years at study initiation.
Wild animals	No wild animals were used in this study.
Reporting on sex	All animals used in these studies were male.
Field-collected samples	No field samples were collected in this study.
Ethics oversight	All animals were handled in accordance with the standards of the Association for the Assessment and Accreditation of Laboratory Animal Care (AAALAC) in an AAALAC-accredited facility (OLAW, Animal Welfare Assurance A4149-01 for NIH). All animal care and procedures were carried out under protocols approved by the NCI Animal Care and Use Committee (ACUC) prior to study initiation.

Note that full information on the approval of the study protocol must also be provided in the manuscript.

Plants

Seed stocks	n/a
Novel plant genotypes	n/a
Authentication	n/a

Flow Cytometry

Plots

Confirm that:

☒ The axis labels state the marker and fluorochrome used (e.g. CD4-FITC).

☒ The axis scales are clearly visible. Include numbers along axes only for bottom left plot of group (a 'group' is an analysis of identical markers).

☒ All plots are contour plots with outliers or pseudocolor plots.

☒ A numerical value for number of cells or percentage (with statistics) is provided.

Methodology

Sample preparation

Sample preparation is described in "online methods" section.

Freshly collected rectal biopsies were digested with collagenase (2 mg/ml; Sigma-Aldrich) in the absence of FBS in 370 C for 1 hour, then it was mechanically separated by using a 10ml syringe with a blunt head cannula. It was washed with R10 and pass through 70um cell strainer. Single cells were counted and used for the experiment.

EDTA whole blood was layered on the top of Ficoll Plaque (GE Healthcare, Chicago, Illinois, USA), and centrifuged at

	2800rmp, 30 minutes, accelerator 4 and decelerator 4. The white PBMC band was collected, washed with PBS and used for experiments.
Instrument	Cytometry acquisition was done using the FACS Symphony A5.
Software	Acquisition was done using FACSDiva software (BD Biosciences, San Jose, California, USA). Cytometry data were analysed using Flow Jo LLC, V10.10
Cell population abundance	No cell sorting experiment was performed.
Gating strategy	NK/ILC gating<br>Singlets, Live cells, CD45+, CD3-CD20-CD11b-, NKp44+/NKG2A+/NKp44-NKG2A-<br><br>Mucosal pDC/mDC gating<br>Singlets, Live cells, CD45+, CD3-CD20-, HLA-DR+, CD14-. Then based on CD11c and CD1623expression different dendritic cell population was defined. CD123+CD11c- is pDC and CD123-CD11c+ is mDC.<br><br>Th1, Th2 and Th17<br>Gating was done on live CD3+CD4+ cells and on vaccine induced Ki67+ cells. CXCR3 and CCR6 expression were used to identify Th1, Th2 or Th17 populations. CXCR3-CCR6- is Th2, CXCR3+CCR6- is Th1 cells and CXCR3-CCR6+ is Th17

☒ Tick this box to confirm that a figure exemplifying the gating strategy is provided in the Supplementary Information.
